# Methyl Syringate Stimulates Glucose Uptake by Inhibiting Protein Tyrosine Phosphatases Relevant to Insulin Resistance

**DOI:** 10.3390/life13061372

**Published:** 2023-06-12

**Authors:** Dohee Ahn, Jihee Kwon, Songyi Song, Jooyoung Lee, Sunyoung Yoon, Sang J. Chung

**Affiliations:** 1 School of Pharmacy, Sungkyunkwan University, Suwon 16419, Republic of Korea; 2 Department of Biopharmaceutical Convergence, Sungkyunkwan University, Suwon 16419, Republic of Korea; 3 Department of Cosmetic Science, Kwangju Women’s University, Gwangju 62396, Republic of Korea

**Keywords:** protein tyrosine phosphatases (PTPs), PTPN2, PTPN6, methyl syringate, catalytic activity, type 2 diabetes, glucose uptake

## Abstract

Several protein tyrosine phosphatases (PTPs), particularly PTPN1, PTPN2, PTPN6, PTPN9, PTPN11, PTPRS, and DUSP9, are involved in insulin resistance. Therefore, these PTPs could be promising targets for the treatment of type 2 diabetes. Our previous studies revealed that PTPN2 and PTPN6 are potential antidiabetic targets. Therefore, the identification of dual-targeting inhibitors of PTPN2 and PTPN6 could be a potential therapeutic strategy for the treatment or prevention of type 2 diabetes. In this study, we demonstrate that methyl syringate inhibits the catalytic activity of PTPN2 and PTPN6 in vitro, indicating that methyl syringate acts as a dual-targeting inhibitor of PTPN2 and PTPN6. Furthermore, methyl syringate treatment significantly increased glucose uptake in mature 3T3-L1 adipocytes. Additionally, methyl syringate markedly enhanced phosphorylation of adenosine monophosphate-activated protein kinase (AMPK) in 3T3L1 adipocytes. Taken together, our results suggest that methyl syringate, a dual-targeting inhibitor of PTPN2 and PTPN6, is a promising therapeutic candidate for the treatment or prevention of type 2 diabetes.

## 1. Introduction

Type 2 diabetes is metabolic disease that affects more than 90% of diabetic patients and is characterized by abnormally high blood sugar levels and insulin resistance in target organs such as adipose tissues, liver, and skeletal muscle [1,2]. Insulin, sulfonylureas, and thiazolidinediones have been used to treat type 2 diabetes [3,4,5,6]. Since long-term treatment with these medications lead to negative effects such as weight gain, cardiovascular complications, and edema, identifying new antidiabetic agents with fewer side effects than commonly used drugs could be a promising approach [7,8]. There is also a need to develop antidiabetic drugs that target multiple proteins to treat type 2 diabetes, as single-targeted therapies have been shown to fail to control blood glucose levels and other comorbidities [9]. Protein tyrosine phosphorylation is associated with various cellular functions, such as proliferation, differentiation, migration, survival, apoptosis, and metabolism, and is regulated by protein tyrosine kinases and protein tyrosine phosphatases (PTPs) [10,11,12]. PTPs regulate cellular protein tyrosine phosphorylation levels by hydrolytically removing phosphate groups from the tyrosine residues of target proteins [13]. Due to their crucial role in cellular processes, dysregulation of PTPs has been linked to various human diseases, including cancer, diabetes, obesity, and autoimmune diseases, suggesting that PTPs may be potential targets for drug development [14,15]. In particular, several PTPs, including PTPN1, PTPN2, PTPN6, PTPN9, PTPN11, PTPRS, and DUSP9, have been reported to interfere with the insulin signaling pathway and induce insulin resistance in type 2 diabetes, suggesting that PTPs are promising targets for the treatment or prevention of type 2 diabetes [16]. Adenosine-monophosphate-activated protein kinase (AMPK), a cellular energy-sensing protein, regulates glucose homeostasis, and its activation stimulates glucose uptake by leading to the translocation of glucose transporter type 4 (GLUT4), suggesting that AMPK is a marker for antidiabetic effect [17,18,19]. We previously showed that knockdown of protein tyrosine phosphatase, i.e., non-receptor type 6 (PTPN6, also named SHP1), increases AMPK phosphorylation in 3T3-L1 adipocytes, suggesting that PTPN6 is a potential antidiabetic target [20]. PTP nonreceptor type 2 (PTPN2, also called TCPTP) downregulates the insulin signaling pathway in vivo by dephosphorylating the insulin receptor [21]. Previously, we identified PTPN2 as an antidiabetic target and discovered its inhibitor, nepetin [22]. Since PTPs relevant to insulin resistance are promising antidiabetic targets, the identification of PTPN2 and PTPN6 inhibitors could be an effective strategy for treating or preventing type 2 diabetes. We found that methyl syringate inhibits the catalytic activity of PTPN2 and PTPN6 in vitro, indicating that methyl syringate targets PTPN2 and PTPN6. Methyl syringate, a polyphenol, is known to have health-promoting properties including antioxidant and antiproliferative effects [23]. According to a report, methyl syringate reduces food intake and delays gastric emptying, suggesting that it contributes to weight suppression [24]. In addition, methyl syringate suppresses hypoxia-induced cyclooxygenase-2 in lung cancer cells, indicating that it may effectively suppress hypoxia-induced inflammation [25]. However, the antidiabetic effects of methyl syringate have not been investigated yet. In this study, we examined the antidiabetic properties of methyl syringate in 3T3-L1 adipocytes. Our study provides evidence for the first time that methyl syringate exerts its antidiabetic effect through a PTP-inhibitory mechanism, and it could be a promising therapeutic candidate for the treatment or prevention of type 2 diabetes.

## 2. Materials and Methods

### 2.1. Overexpression and Purification of PTPN2 and PTPN6

The methods used for overexpressing and purifying PTPN2 and PTPN6 have been described previously [20,22]. Recombinant plasmids carrying PTPN2 and PTPN6 were transformed into *Escherichia* (*E.*) *coli* Rosetta (DE3) cells (Merck KGaA, Darmstadt, Germany). For the expression of recombinant PTPN2 or PTPN6, cells were induced by adding 0.1 mM isopropyl-1-thio-β-D-galactoside for 16 h at 18 °C. The cells were then centrifuged at 2691× *g* for 15 min at 4 °C, resuspended in lysis buffer, and lysed by ultrasonication. The cells were centrifuged, and the supernatant was maintained with cobalt affinity resin (TALON^®^; Takara Korea, Seoul, South Korea). After washing the resin with lysis buffer containing 10 mM imidazole, PTPN2 and PTPN6 were eluted using lysis buffer supplemented with 100 mM imidazole.

### 2.2. Measurement of Catalytic Activity, Half-Inhibitory Concentration (IC_50_) Values, and Hill Coefficient

The enzymatic activities of PTPN2 and PTPN6 were determined using a commonly used PTP substrate, 6,8-difluoro-4-methylumbelliferyl phosphate (DiFMUP). The methods used for measuring catalytic activity have been described previously [20,22]. To determine the kinetic constants, 2 nM PTPN2 or 6 nM PTPN6 was mixed with various concentrations of DiFMUP in the reaction buffer. The fluorescence intensity was evaluated, and kinetic constants such as *K*_M_, *V*_max_, and *k*_cat_ were obtained using Michaelis–Menten plots and Lineweaver–Burk plots. To measure the half-inhibitory concentration (IC_50_) values of methyl syringate against PTPN2 or PTPN6, various concentrations of methyl syringate (200, 100, 50, 25, 5, 1, and 0.1 µM for PTPN2 and 320, 160, 40, 20, 10, 1, and 0.1 µM for PTPN6) were added to DiFMUP at a concentration of 2 × *K*_M_ (61 µM for PTPN2 and 843 µM for PTPN6) and mixed with PTPN2 or PTPN6. To estimate the IC_50_ values, nonlinear regression analysis was carried out using GraphPad Prism 5 software (GraphPad Software Inc., San Diego, CA, USA). The Hill coefficient (n_H_), which shows the cooperativity between methyl syringate and PTPs, was obtained as the slope of the Hill plot using the Hill equation [26].

### 2.3. Cell Culture

3T3-L1 preadipocytes were purchased from Zen-Bio, Inc. (Research Triangle Park, NC, USA). The cells were maintained with high-glucose Dulbecco’s modified Eagle’s medium (DMEM; Welgene Inc., Gyeongsan-si, South Korea) consisting of 10% bovine calf serum (Welgene) and an antibiotic-antimycotic solution (Welgene) at 37 °C in a 5% CO_2_ incubator.

### 2.4. Cell Differentiation

After the 3T3-L1 preadipocytes reached 100% confluency, cells were maintained for two days in DMEM containing 0.5 mM isobutylmethylxanthine (Merck KGaA), 1 μM dexamethasone (Sigma-Aldrich, Saint Louis, MO, USA), 5 μg/mL insulin (Merck KGaA), 10% fetal bovine serum (FBS; Welgene), and an antibiotic-antimycotic solution (Welgene). The cells were then incubated for two days in DMEM consisting of 5 μg/mL insulin (Merck KGaA), 10% FBS (Welgene), and antibiotic-antimycotic solution (Welgene). Subsequently, the cells were maintained for two days in DMEM containing 10% FBS (Welgene) and an antibiotic-antimycotic solution and incubated at 37 °C in a 5% CO_2_ incubator.

### 2.5. RNA Interference

Knockdown of PTPN2 and PTPN6 in 3T3-L1 preadipocytes was achieved using small interfering RNAs (siRNAs). The PTPN2 siRNA was obtained from Bioneer (Daejeon, South Korea), while PTPN6 siRNA and scrambled siRNA (control siRNA) were purchased from Integrated DNA Technologies Inc. (San Diego, CA, USA). 3T3-L1 preadipocytes were transfected with PTPN2 siRNA (20 nM), PTPN6 siRNA (2 nM), or scrambled siRNA (control siRNA) for 48 h using Dharmafect transfection reagent (Dharmacon, GE Healthcare Korea, Songdo, Korea) following the manufacturer’s instructions. The efficiency of PTPN2 and PTPN6 knockdown was evaluated using quantitative real-time–polymerase chain reaction (qRT-PCR).

### 2.6. Quantitative Real-Time–Polymerase Chain Reaction (qRT-PCR)

The methods used for qRT-PCR have been described previously [20]. Total RNA was extracted from 3T3-L1 cells using AccuPrep^®^ Universal RNA Extraction Kit (Bioneer), and 1 µg of total RNA was used to synthesize cDNA using a High-Capacity Reverse Transcription kit (Applied Biosystems, Foster City, California, USA). PCR was carried out on a CFX Connect Real-Time PCR Detection System (Bio-Rad, Hercules, CA, USA) using SsoAdvanced Universal SYBR Green Supermix (Bio-Rad) according to the manufacturer’s instructions. The gene expression levels of PTPN2 and PTPN6 were normalized to the expression levels of the control gene, GAPDH. The primer sequences for each gene are provided in Table 1.

### 2.7. Cell Viability Assay

Differentiated 3T3-L1 adipocytes were incubated in low-glucose DMEM (Gibco BRL, Middlesex, UK) for 16 h. Subsequently, the cells were treated with 5, 10, or 20 μM methyl syringate in glucose-depleted DMEM (Gibco BRL) for 6 h, and cell viability was measured using an EZ-Cytox cell viability assay kit (Daeil Lab Service, Seoul, South Korea) according to the manufacturer’s instructions. The absorbance was measured at 450 nm using a microplate reader (Victor^TM^ X4, Perkin Elmer, Waltham, MA, USA).

### 2.8. Glucose Uptake Assay

The methods used for measuring glucose uptake have been described previously [1,22]. Differentiated 3T3-L1 adipocytes were cultured in low-glucose DMEM (Gibco BRL) for 16 h. The cells were then treated with methyl syringate for 6 h or insulin (positive control) for 30 min in glucose-depleted DMEM (Gibco BRL). Subsequently, cells were incubated with a fluorescent glucose probe, 100 μM 2-[N-(7-nitrobenz-2-oxa-1,3-diazol-4-yl)amino]-2-deoxyglucose (2-NBDG; Thermo Fisher Scientific, Waltham, MA, USA), for 1 h, and the fluorescence intensity was measured using a fluorescence microplate reader (Victor^TM^ X4).

### 2.9. Western Blotting

The methods used for Western blotting have been described previously [2,22]. Proteins were separated by electrophoresis and transferred to a polyvinylidene fluoride membrane (Merck KGaA) via a wet transfer system. The membranes were then maintained overnight at 4 °C with the following primary antibodies: anti-total AMPK, anti-phosphorylated AMPK (Cell Signaling Technology, Beverly, MA, USA), and anti-β-actin (AbFrontier, Seoul, South Korea). The membranes were then maintained with an anti-rabbit IgG-horseradish peroxidase-conjugated secondary antibody (Santa Cruz Biotechnology, Dallas, TX, USA) for 1 h at room temperature. Antibody-antigen complexes were visualized using the EzWestLumi Plus Detection Kit (ATTO Corporation, Tokyo, Japan). Luminescent images were obtained using a LuminoGraph II Imaging System (ATTO Corporation).

### 2.10. Oil Red O Staining

Lipid droplets in 3T3-L1 cells were observed by Oil Red O staining as described previously [27,28]. 3T3-L1 preadipocytes were differentiated in the presence of methyl syringate, and on day 6 after differentiation, the cells were washed with PBS and fixed with 4% paraformaldehyde for 15 min. After washing the cells with PBS, the cells were stained with filtered 0.3% Oil Red O solution in isopropanol for 30 min. Images of the lipid droplets were obtained using a Cytation 7 cell-imaging multimode reader (BioTek, Winooski, VT, USA). To quantify lipid accumulation, the Oil Red O dye was eluted by incubation with 100% isopropanol for 30 min, and the absorbance was evaluated at 490 nm using a microplate reader (Victor^TM^ X4).

### 2.11. Statistical Analysis

Statistical significance (*p* < 0.05) was assessed using two-tailed unpaired *t*-test in GraphPad (GraphPad Software, San Diego, CA, USA). All experiments were performed independently three times.

## 3. Results and Discussion

### 3.1. Suppression of PTPN2 and PTPN6 Increased AMPK Phosphorylation

**Figure 1:**
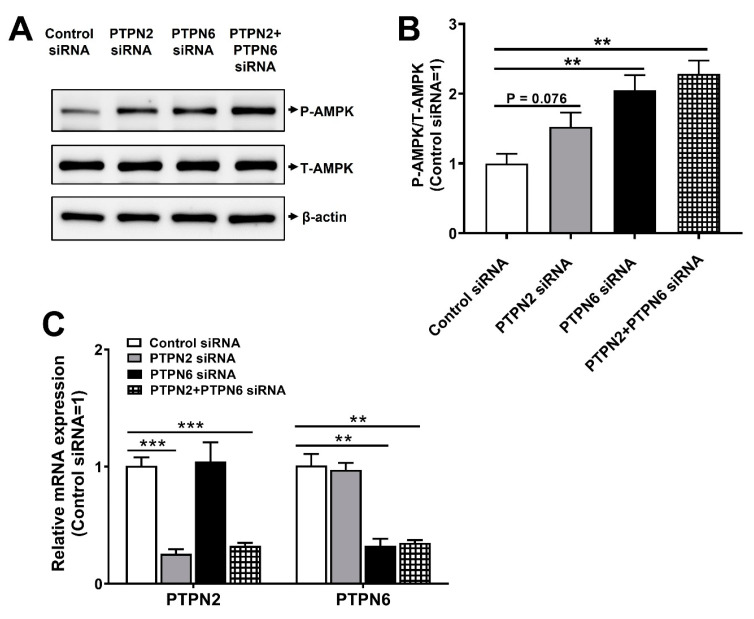
Suppression of PTPN2 and PTPN6 enhances phosphorylation. (**A**–**C**) 3T3-L1 preadipocytes were transfected with PTPN2, PTPN6 siRNAs, or scrambled siRNA (control siRNA). After 48 h, cells were lysed and analyzed using Western blotting (**A**,**B**) or quantitative real-time PCR (**C**). (**B**) Quantification of phospho-AMPK and total-AMPK using ATTO image analysis software. Results are expressed as mean ± standard error of the mean (SEM). Data were analyzed using a two-tailed unpaired *t*-test. *** *p* < 0.001; ** *p* < 0.01 compared to the control siRNA.

Since some PTPs, including PTPN1, PTPN2, PTPN6, PTPN9, PTPN11, PTPRS, PTPRF, and DUSP9, have been reported to weaken insulin action and induce insulin resistance associated with type 2 diabetes, these PTPs could be potential therapeutic targets to treat or prevent type 2 diabetes [13,14]. In previous studies, we identified PTPN2 and PTPN6 as potential antidiabetic targets [20,22]. In this study, we performed knockdown of PTPN2 and PTPN6 in 3T3-L1 preadipocytes using siRNAs and observed the phosphorylation levels of AMPK by Western blotting. AMPK is relevant for the maintenance of glucose homeostasis, and its activation is associated with improved glucose uptake in skeletal muscles and adipose tissues, suggesting that AMPK is a marker of antidiabetic properties [18,19,29]. Consistent with our previous findings [20,22], we observed that the inhibition of PTPN2 or PTPN6 resulted in increased AMPK phosphorylation. This suggests that targeting these PTPs could be a promising therapeutic strategy for treating or preventing type 2 diabetes (Figure 1A,B). Furthermore, simultaneous knockdown of both PTPN2 and PTPN6 synergistically enhanced AMPK phosphorylation, suggesting that these PTPs could be a good pair for identifying dual-targeting inhibitors (Figure 1A,B). Efficient suppression of PTPN2 and PTPN6 expression was confirmed using qRT-PCR (Figure 1C).

### 3.2. Methyl Syringate Inhibited the Catalytic Activity of PTPN2 and PTPN6 In Vitro

**Figure 2:**
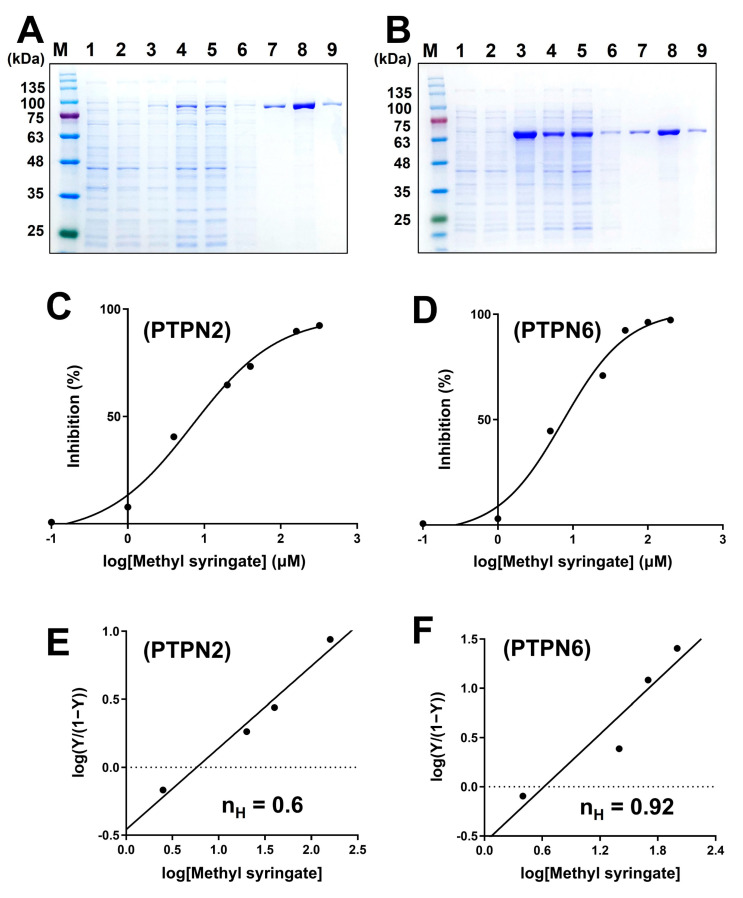
Methyl syringate inhibits the catalytic activity of PTPN2 and PTPN6. (**A**,**B**) PAGE analysis of PTPN2 ((**A**) molecular weight: 89.7 kDa) and PTPN6 ((**B**) molecular weight: 67.6 kDa). M, molecular weight marker; lane 1, total sample before induction; lane 2, supernatant before induction; lane 3, total sample after induction; lane 4, supernatant after induction; lane 5, sample passed through the column after sonication; lane 6, sample after washing the column using lysis buffer; lane 7, sample after washing the column using 10 mM imidazole’ lanes 8 and 9, protein eluted using 100 mM imidazole. (**C**,**D**) IC_50_ values for methyl syringate were estimated using a sigmoid curve against percent inhibition (%) for log[Methyl syringate] (μM). **(E**,**F**) The Hill coefficient (n_H_) was obtained from slopes of Hill plots using the Hill equation.

Since PTPN2 and PTPN6 negatively regulate insulin action, they are considered potential drug targets for treating type 2 diabetes, and their inhibitors have been shown to have antidiabetic properties [20,22]. In this study, we observed that suppression of PTPN2 and PTPN6 stimulated AMPK phosphorylation, suggesting that PTPN2 and PTPN6 are promising antidiabetic targets. Hence, the identification of a dual-targeting inhibitor of PTPN2 and PTPN6 could be a promising approach to treat or prevent type 2 diabetes. In this study, PTPN2 and PTPN6 were overexpressed in *E. coli* and subsequently purified using a cobalt-affinity resin, as shown in Figure 2A,B. To estimate the catalytic activities of these PTPs, their kinetic constants were obtained (Table 2). Subsequently, we found that methyl syringate inhibited the catalytic activity of PTPN2 and PTPN6, and the half-inhibitory concentration (IC_50_) values of methyl syringate for PTPN2 and PTPN6 were determined to be 6.95 and 7.31 μM, respectively (Figure 2C,D). In addition, methyl syringate showed better inhibition of DiFMUP hydrolysis by PTPN2 and PTPN6 than other PTPs such as PTPN3, PTPN5, PTPN14, PTPRS, PTPRG, and PTPRN (Supplementary Figure S1). Next, we assessed the Hill coefficient (n_H_) to examine the cooperativity between methyl syringate and PTPs. The n_H_ values obtained from the slope of the Hill plot display the degree of cooperativity of the ligand–protein interactions (n_H_ > 1, positive cooperative binding; n_H_ = 1, non-cooperative binding; n_H_ < 1, negative cooperative binding) [26]. The n_H_ values of methyl syringate for PTPN2 and PTPN6 were obtained as 0.60 and 0.92, respectively, demonstrating a negative cooperativity in the binding of methyl syringate to PTPs (Figure 2E,F). These results indicate that methyl syringate acts as a dual-targeting inhibitor of PTPN2 and PTPN6.

### 3.3. Glucose Uptake Is Enhanced following Methyl Syringate Treatment

**Figure 3:**
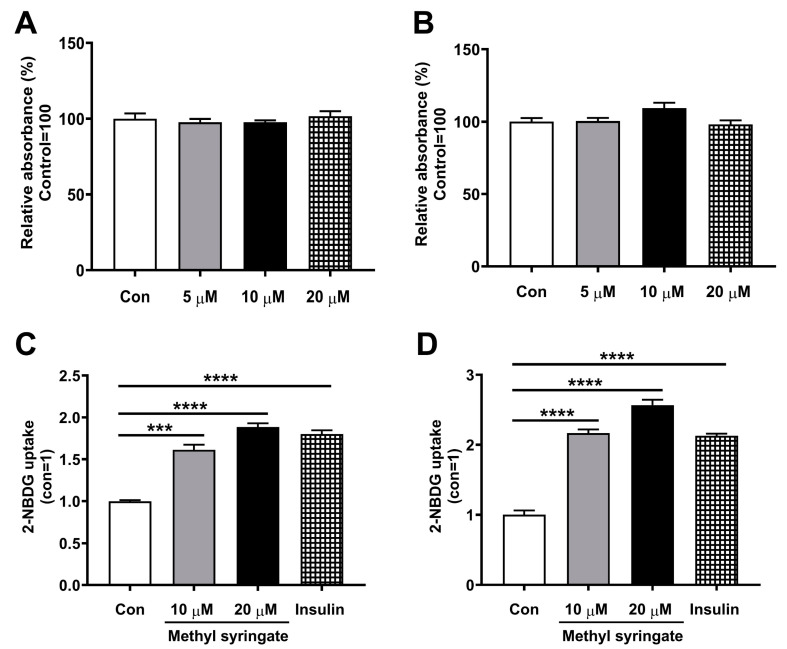
Methyl syringate enhances glucose uptake. (**A**) Differentiated 3T3-L1 adipocytes were incubated with low-glucose DMEM for 16 h. Subsequently, the cells were then treated with 5, 10, or 20 μM methyl syringate in glucose-depleted DMEM for 6 h, and cell viability was assessed. (**B**) 3T3-L1 preadipocytes were differentiated in the presence of 5, 10, or 20 μM methyl syringate for 3 days, and cell viability was evaluated. (**C**,**D**) Differentiated 3T3-L1 adipocytes (**C**) and C2C12 muscle cells (**D**) were incubated with 10 and 20 μM methyl syringate, control (0.1% dimethyl sulfoxide treatment group), or 0.1 μM insulin (positive control) for 6 h (methyl syringate and control) or 30 min (insulin). Next, the cells were maintained with the fluorescent glucose indicator 2-NBDG for 1 h, and the fluorescence intensity was measured. Results are presented as mean ± SEM. Data were analyzed using a two-tailed unpaired *t*-test. **** *p* < 0.0001; *** *p* < 0.001 compared to the control group.

Considering that glucose uptake by insulin-sensitive tissues such as skeletal muscles, liver, and adipocytes leads to the maintenance of glucose homeostasis [30,31,32], we next investigated the antidiabetic effects of methyl syringate on glucose uptake. We previously reported that several natural compounds exhibit antidiabetic properties by inhibiting PTPs associated with insulin resistance [2,22,28,33,34]. To determine the appropriate concentration of methyl syringate for treatment of 3T3-L1 adipocytes, cell viability was evaluated. Incubation of differentiated 3T3-L1 adipocytes with 5, 10, or 20 μM methyl syringate for 6 h did not influence cell viability, indicating that these concentrations were appropriate for cell treatment (Figure 3A). In addition, 3T3-L1 preadipocytes were differentiated in the presence of 5, 10, or 20 μM methyl syringate for 3 days, and cell viability was evaluated. Importantly, incubation with methyl syringate did not affect cytotoxicity of the cells (Figure 3B). Next, we investigated the effect of methyl syringate on glucose uptake by differentiated 3T3-L1 adipocytes and C2C12 muscle cells. Cells were treated with 10 or 20 μM methyl syringate, 0.1 μM insulin (positive control), or control (0.1% dimethyl sulfoxide treatment group) for 6 h (methyl syringate and control) or 30 min (insulin). The cells were then incubated with 2-NBDG for 1 h, and the fluorescence intensity was assessed. Incubation with methyl syringate significantly enhanced fluorescence intensity compared with the control, indicating that methyl syringate stimulated glucose uptake in differentiated 3T3-L1 adipocytes and C2C12 muscle cells (Figure 3C,D). Insulin was used as a positive control because it promotes glucose uptake in skeletal muscles and adipose tissues [35]. In addition, treatment with 0.1 μM insulin markedly increased glucose uptake compared to the control group, indicating that the fluorescent glucose probe functioned properly in cellular systems (Figure 3C,D).

### 3.4. Methyl Syringate Increased AMPK Phosphorylation in 3T3-L1 Adipocytes

**Figure 4:**
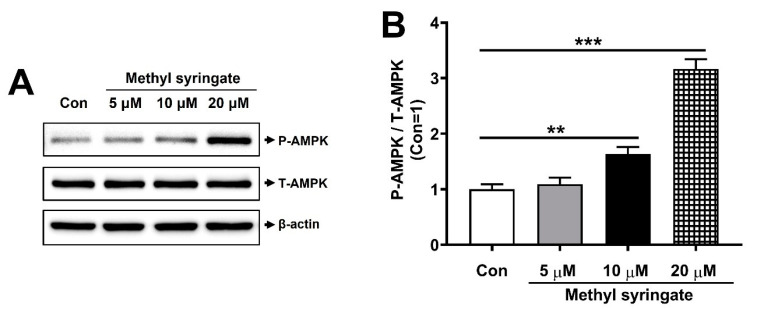
Methyl syringate increases AMPK phosphorylation. (**A**) 3T3-L1 adipocytes were treated with of 5, 10, and 20 μM methyl syringate for 6 h, and Western blotting was carried out using antibodies against phosphorylated AMPK, total AMPK, and β-actin. (**B**) Quantification of phospho-AMPK/total-AMPK using the ATTO image analysis software. Results are presented as mean ± SEM. Data were analyzed using a two-tailed unpaired *t*-test. *** *p* < 0.001; ** *p* < 0.01 compared to the control group.

AMPK is a metabolic regulator that plays an important role in controlling glucose homeostasis, and its activation leads to the stimulation of glucose uptake by promoting GLUT4 translocation [18,19,29]. Metformin, an oral antidiabetic drug, has been shown to improve AMPK activation, indicating that AMPK is a marker for antidiabetic properties [36,37,38]. We investigated whether methyl syringate enhances AMPK phosphorylation in mature 3T3-L1 adipocytes by incubating them with 5, 10, or 20 μM methyl syringate for 6 h, followed by Western blotting. Our study showed that the treatment with 10 or 20 μM methyl syringate significantly increased AMPK phosphorylation compared to the control (Figure 4A,B). These results indicate that methyl syringate, a dual-targeting inhibitor of PTPN2 and PTPN6, stimulated glucose uptake through AMPK activation. Further studies investigating whether methyl syringate induces translocation of GLUT4 in cell-based studies would be interesting.

### 3.5. Methyl Syringate Did Not Enhance Lipid Accumulation

**Figure 5:**
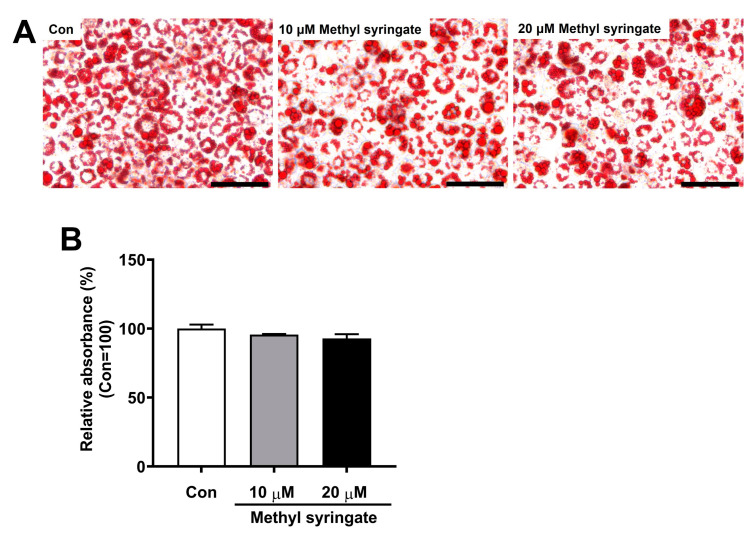
Methyl syringate does not enhance lipid accumulation. (**A**) 3T3-L1 preadipocytes were differentiated into mature adipocytes in the presence of 10 or 20 μM methyl syringate. Lipid droplets was evaluated by Oil Red O staining on day 6 after differentiation. (**B**) To quantify lipid accumulation, Oil Red O dye was eluted by treatment with isopropanol, and the absorbance was measured at 490 nm using a microplate reader. Results are presented as mean ± SEM. Scale bars: 100 μm.

We investigated the effects of methyl syringate on lipid accumulation since drugs used to treat type 2 diabetes, such as insulin, sulfonylureas, and thiazolidinediones, are associated with weight gain [3,4,5,6]. Weight gain has been associated with worsened blood sugar control and increased risk of cardiovascular disease [3,6]. In this study, we differentiated 3T3-L1 preadipocytes in the presence of methyl syringate and performed Oil Red O staining. On day 6 of differentiation, we evaluated the degree of lipid droplet formation and quantified lipid accumulation. Our results showed that methyl syringate did not affect adipocyte differentiation compared to the control, indicating that it did not induce lipid accumulation (Figure 5A,B). These results suggest that methyl syringate improves glucose uptake without weight gain as a side effect.

## 4. Conclusions

As PTPN2 and PTPN6 have been shown to negatively regulate insulin action, the identification of inhibitors of these proteins is considered an effective strategy for the treatment or prevention of type 2 diabetes. Our study demonstrated that the suppression of PTPN2 and PTPN6 resulted in enhanced AMPK phosphorylation, indicating that targeting PTPN2 and PTPN6 could be a promising approach for the development of antidiabetic targets. We showed that methyl syringate inhibits the catalytic activity of PTPN2 and PTPN6, indicating that it targets these PTPs. Furthermore, methyl syringate treatment stimulated glucose uptake via AMPK activation in mature 3T3-L1 adipocytes. In conclusion, our results suggest that methyl syringate, a dual-targeting inhibitor of PTPN2 and PTPN6, exhibits antidiabetic effects and can be used as a functional food ingredient or pharmaceutical supplement to prevent type 2 diabetes.

## Figures and Tables

**Table 1:** Sequences of primers for mouse genes.

Gene Name	Primer Sequence
GAPDH	
forward	GGC AAA TTC AAC GGC ACA GT
reverse	GGC GGA GAT GAT GAC CCT TT
PTPN2	
forward	TGG CAG CCG TTA TAC TTG GA
reverse	CAG CAT GTG TTC GGA AGT GG
PTPN6	
forward	CTT GGC AGG AGA ACA CTC GT
reverse	GCC ATA TCT CCC GAA CCA GG

**Table 2:** Kinetic constants of DiFMUP hydrolysis by PTPN2 and PTPN6.

	[E] (nM)	*K*_M_ (µM)	*V*_max_ (µMmin^−1^)	*k*_cat_ (min^−1^)	*k*_cat_/*K*_M_ (µM^−1^ min^−1^)
PTPN2	2	30.46	0.72	3.6 × 10^2^	11.8
PTPN6	6	421.3	6.23	1.03 × 10^3^	2.46

## Data Availability

All data have been provided in the manuscript. Detailed methods and additional data are available from the corresponding author upon request.

## Supplementary Materials

The following supporting information can be downloaded at: https://www.mdpi.com/article/10.3390/life13061372/s1, Figure S1: Inhibition of PTPs by methyl syringate treatment. PTPs were added to solutions containing 20 µM methyl syringate in reaction buffer with DiFMUP (2 × *K*_M_) and catalytic activity was measured.
