# Supplementary material for: Methyl Syringate Stimulates Glucose Uptake by Inhibiting Protein Tyrosine Phosphatases Relevant to Insulin Resistance
Source: Life (Basel). 2023 Jun 12;13(6):1372. doi: 10.3390/life13061372 (PMC10302301; doi:10.3390/life13061372)
Supplement: Supplementary file 1 [file life-13-01372-s001.zip › life-2397197-supplementary.pdf]

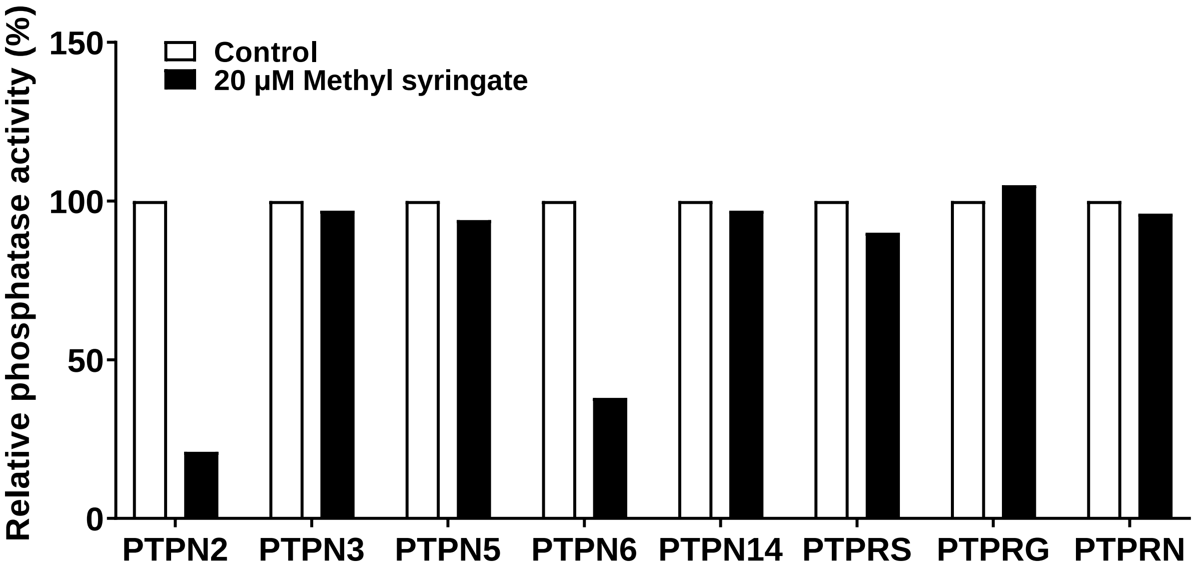

Supplementary Figure S1. Inhibition of PTPs by methyl syringate treatment. PTPs were added to solutions containing 20 μM methyl syringate in reaction buffer with DiFMUP (2 × K_M) and catalytic activity was measured.
